# Rocky Mountain Spotted Fever in Dogs, Brazil

**DOI:** 10.3201/eid1503.081227

**Published:** 2009-03

**Authors:** Marcelo B. Labruna, Orson Kamakura, Jonas Moraes-Filho, Mauricio C. Horta, Richard C. Pacheco

**Affiliations:** University of São Paulo, São Paulo, Brazil (M.B. Labruna, J. Moraes-Filho, M.C. Horta, R.C. Pacheco); Instituto Dog Bakery de Medicina Animal, São Paulo (O. Kamakura)

**Keywords:** Rickettsia rickettsii, dogs, Rocky Mountain spotted fever, Brazil, dispatch

## Abstract

Clinical illness caused by *Rickettsia rickettsii* in dogs has been reported solely in the United States. We report 2 natural clinical cases of Rocky Mountain spotted fever in dogs in Brazil. Each case was confirmed by seroconversion and molecular analysis and resolved after doxycycline therapy.

*Rickettsia rickettsii,* the etiologic agent of Rocky Mountain spotted fever (RMSF), is the most pathogenic of the rickettsiae for humans and some animals. RMSF has been reported in North, Central, and South America, where different tick species serve as vectors (*1*). Although serologic studies among healthy dogs in Brazil have indicated past infection by *R. rickettsii* (*2*,*3*), clinical illness caused by *R. rickettsii* in dogs has been reported solely in the United States (*4*,*5*).

In Brazil, the most common vector-borne disease of dogs is canine monocytic ehrlichiosis (CME), caused by *Ehrlichia canis* (*6*). Clinical signs (fever, depression, petechial hemorrhages, thrombocytopenia) in dogs with overt RMSF infection or CME are often similar (*5*). Doxycycline is the treatment of choice for *R. rickettsii* infection in dogs (*7*) and the most commonly prescribed treatment for CME in Brazil. Thus, clinical cases of RMSF among dogs in Brazil could be being misdiagnosed as CME. We describe 2 natural cases of RMSF in dogs in Brazil.

## The Cases

On August 23, 2007, a 4-year-old, female, Dogue de Bordeaux (dog 1) was brought to a veterinary clinic in São Paulo because of a high load of ticks noticed 5 days after she had been to a farm in the Itu Municipality (23°15′S, 47°17′W), state of São Paulo. The dog was treated with fipronil and sent home. Tick taxonomic identification was not performed. The next day, the dog had diarrhea and hematochezia and was taken back to the clinic, where laboratory test results were within reference ranges, except for a slight leukocytosis (18,000 cells/mm^3^) and elevated alkaline phosphatase level (278.6 U/L). Metronidazol was prescribed, and the dog was again sent home. Three days later, the dog was febrile (40.5°C), anorexic, and lethargic. Blood was sent to a private laboratory, where a battery of PCR tests failed to detect DNA of *Babesia* spp., *Borrelia* spp., *Mycoplasma* spp., or *Ehrlichia* spp. The dog was treated with subcutaneous imidocarb and oral doxycycline. The next day, the dog was still febrile (39.4°C) and anorexic, and neurologic signs (ataxia and vestibular syndrome with spontaneous nystagmus) had developed. The animal was hospitalized; doxycycline was switched to the subcutaneous route; and the next day oral prednisone was added. Blood values remained within reference range, except for a slight leukocytosis (17,600 cells/mm^3^). On August 30, neurologic improvement was noted, and the dog had no fever (38.5°C) and started to eat. Despite slight nystagmus, the dog was discharged the next day. On September 3, (8 days after doxycycline therapy began), the dog showed no clinical abnormality, and a new blood sample was collected for serologic testing. Another blood sample collected on September 10 showed hematologic parameters within reference range except for leukopenia (6,900 cells/mm^3^).

Serologic evaluation was performed by indirect immunofluorescence assay (IFA) by using antigens of 6 *Rickettsia* isolates from Brazil (*8*). Plasma from the sample collected on August 24 showed an IFA endpoint titer of 128 for *R. rickettsii* and no reactivity for the remaining rickettsial antigens at a 1:64 dilution. Plasma from the sample collected on September 3 showed the following endpoint titers for rickettsial antigens: *R. rickettsii* 2,048, *R. parkeri* 512, *R. amblyommii* 512, *R. felis* 512, *R. rhipicephali,* and 512; *R. bellii* 256.

DNA was extracted from the blood samples collected on August 24 and September 3 (before and after antimicrobial drug therapy) by using the DNeasy Tissue Kit (QIAGEN, Chatsworth, CA, USA). Samples were tested by 2 PCR protocols: one targeting a 147-bp fragment of the rickettsial *gltA* gene (*9*), and the other, a heminested PCR, targeting a fragment of the rickettsial *ompA* gene (*10*). Extracted DNA from the first blood sample yielded expected products by both PCR protocols. No product was obtained from the second blood sample. Sequencing of the *ompA* product resulted in a 452-bp fragment 100% identical to the corresponding sequence of the Bitterroot strain of *R. rickettsii* from the United States (GenBank accession no. U43804). *Ehrlichia* spp. were not detected by PCR (*6*) in either sample.

The second case was noted on August 28, 2007, when a 10-month-old, female, miniature Schnauzer (dog 2) was examined at the same veterinary clinic for anorexia, lethargy, fever (40.2°C), vomiting, and tick infestation. This dog had visited the same farm at the same time as dog 1. No neurologic signs were observed. Dog 2 was treated with fipronil and sent home with atropine, imizol, ranitidine, dipirone, and doxycycline. Blood collected on August 28 had values within reference ranges, except for thrombocytopenia (thrombocytes 150,000/mm^3^). IFA for rickettsial antigens showed no reactivity at the 1:64 dilution for the 6 rickettsial antigens, but serum from a second sample collected on September 3 (when the dog was showing no clinical signs) showed the following endpoint titers: *R. rickettsii* 4,096 *R. parkeri* 512, *R. amblyommii* 512, *R. felis* 256, *R. rhipicephali* 256, and *R. bellii* 256.

DNA was extracted from the samples collected on August 24 and September 3 (before and after antimicrobial drug therapy) and processed by the PCR protocols cited above. Extracted DNA from the first sample yielded expected product for the *gltA-*PCR, which was not sequenced. No other PCR product was obtained.

## Conclusions

Definitive diagnoses of naturally acquired *R. rickettsii* infection in 2 dogs in Brazil are supported by 1) paired serum samples with >8-fold rise in antibody titer to *R. rickettsii* antigen; 2) titers to *R. rickettsii* >4-fold higher than titers to other rickettsial antigens known to occur in Brazil; 3) detection of rickettsial DNA in canine blood, confirmed to be *R. rickettsii* in at least 1 of the dogs; 4) compatible clinical signs and laboratory abnormalities (i.e., thrombocytopenia in at least 1 dog); 5) response to doxycycline; and 6) compatible epidemiologic history (i.e., prior contact with ticks in an RMSF-endemic area). This sixth statement is supported by the fact that Itu municipality is an area where RMSF laboratory-confirmed cases in humans have been reported since 2003 (www.cve.saude.sp.gov.br). Owners of the 2 dogs reported here noted various capybaras (*Hydrochoerus hydrochaeris*) in the area where their dogs had become infested with ticks (data not shown). Capybaras are one of the main hosts of *Amblyomma cajennense* ticks, the most common important vector of *R. rickettsii* in Brazil (*9*,*11*).

In a recent study of experimental infection, dogs exposed to a Brazil isolate of *R. rickettsii* had fever, lethargy, anorexia, anemia, and thrombocytopenia; 1 also had ocular lesions (*12*). These clinical signs have been reported in the United States for dogs with active *R. rickettsii* infection (*4*,*5*) and were also noted in the present study under natural conditions, except for anemia and ocular lesions. Studies in the United States have shown that neurologic dysfunction occurs in as many as 43% of dogs with RMSF; vestibular dysfunction is possibly the most frequent neurologic abnormality (*13*). These results suggest that clinical illness caused by *R. rickettsii* in dogs has similar patterns in Brazil and the United States.

Veterinarians in Brazil should include *R. rickettsii* infection in their differential diagnoses of CME and other acute nonspecific febrile illnesses of dogs, especially because *R. rickettsii* is highly pathogenic for humans. In the United States, several cases of human infection have been preceded by RMSF in dogs (*14*,*15*). Accurate diagnosis of RMSF in dogs should lead to dog owners understanding risk for infection from ticks in their location (*14*) and provide valuable information for the surveillance of RMSF in humans.
